# Early Intervention, Regular Education, and Family: Reciprocal Influences on Communication and Language Disorders

**DOI:** 10.3390/children11010043

**Published:** 2023-12-29

**Authors:** María Alcalá-Cerrillo, Sabina Barrios-Fernández, Maria Ángeles García-Gil, José Carmelo Adsuar, Florencio Vicente-Castro, Jessica Fernández-Solana, Jerónimo J. González-Bernal

**Affiliations:** 1 Occupation, Participation, Sustainability and Quality of Life (Ability Research Group), Nursing and Occupational Therapy College, University of Extremadura, 10003 Cáceres, Spain; malcala@unex.es (M.A.-C.); sabinabarrios@unex.es (S.B.-F.); 2 Promoting a Healthy Society Research Group (PHeSO), Faculty of Sport Sciences, University of Extremadura, 10003 Cáceres, Spain; mariagarcia@unex.es; 3 Developmental and Educational Psychology of Childhood, Teens, The Elderly and Disabilities Association (INFAD), University of Extremadura, 06006 Badajoz, Spain; fvicente@unex.es; 4 Health Sciences Department, University of Burgos, 09001 Burgos, Spain; jfsolana@ubu.es (J.F.-S.); jejavier@ubu.es (J.J.G.-B.)

**Keywords:** early childhood care, communication disorders, educational intervention, family participation, child development

## Abstract

Families are the primary caregivers and the main source of support for their children. Family resilience involves coping and adapting to stressful situations. This study explored the impact of previous treatment experience on parental resilience, in families, as well as the relationship between family history of communication and language disorders and parental stress. These variables were assessed through the Resilience Scale and the Parental Stress Index in parents of 220 children aged 3 to 6 years attending mainstream schools and early intervention (EI) centers in Caceres (Spain). The results revealed significant differences in resilience between parents who had received previous treatments and those who had not (*p* = 0.11). Furthermore, a significant association was found between having no family history of communication and language disorders and the Dysfunctional Parent–Child Interaction subscale from the Parental Stress Index (*U* = −2.079, *p* = 0.038). These findings highlight the relevance of previous experience in EI to build family resilience as resilient parents are more likely to be actively involved in their children’s education and create a supportive environment. Thus, promoting resilience in educational settings may have positive effects on children’s and families’ quality of life during the EI process.

## 1. Introduction

Early intervention (EI) refers to the systematic intervention process for children with or at risk of developmental disorders from birth to 6 years of age [1]. Currently, according to the White Paper on EI, it is defined as “the set of actions aimed at children aged 0–6, their families, and their environment, with the objective of responding as early as possible to the transient or permanent needs of children with developmental disorders or those at risk of experiencing them. These interventions, which must consider the child’s overall well-being, should be planned by a team of interdisciplinary or transdisciplinary professionals” [2]. Having a child, especially for first-time parents, involves considerable stress and anxiety until the adjustment to the new family member and lifestyle [3]. This is aggravated when the child has been diagnosed with a disorder that may lead to a disability [4,5,6,7]. Research on parents with young children with language delay suggests they experience higher stress levels than parents with typically developed children. These findings have been particularly observed in children at earlier stages of development, particularly in those with poor expressive language [8,9,10]. Likewise, interventions in EI for children with these disorders are gaining particular relevance due to the frustration experienced by children and families caused by the inability to express themselves and comprehend speech. Additionally, the increasing incidence of these cases is contributing to this heightened significance. The incidence is on such a rise that there is a growing number of studies related to the detection, prevention, or intervention of difficulties during language development and acquisition in early childhood [11]. According to various studies, the prevalence of these disorders ranges from 10 to 15% [12], although other researchers indicate that the prevalence in late speech development is around 13% [13]. Furthermore, some studies also demonstrate that between 17 and 26% of children with these disorders may continue to struggle with difficulties up to 6 years of age [14].

Evidence suggests that preschool parents of children with language impairment may experience high levels of parental stress because of feelings of insecurity and helplessness in parenting their children [15,16]. A disorder diagnosis in a child may precipitate significant emotional reactions in parents. Nevertheless, the family’s ability to face and cope with the challenges inherent in this situation may lie within their family unit [17,18].

Families are primary caregivers and supporters when facing difficulties [19]. Higher stress in families with children with communication disorders leads to tensions in relationships compared to parents without a history of communication disorders [20,21] so addressing these challenges is essential to promote healthy family relationships and facilitate effective communication [22,23]. Evidence underlines the importance of family collaboration for the care of children with developmental disorders [24,25,26,27], recognizing the need for the family to be trained on the disorder so that they can work together on the educational and therapeutic goals in the family setting [28,29,30,31]. Thus, parents who have participated in previous EI interventions can exhibit higher resilience compared to those who have not, especially concerning their children with language and communication disorders [32]. In this regard, parental resilience is identified as a protective and positive responsive attitude that strengthens an individual against adverse or risky environments encountered throughout life [33]. Parental resilience plays a fundamental role in the development of these children, as resilient parents tend to better adapt to associated challenges, fostering a more stable and supportive environment for their children. The parents’ ability to overcome stress and adversities linked to these disorders can significantly impact the emotional well-being of the children. Moreover, a resilient attitude on the part of the parents can promote a greater search for support and resources, thus facilitating access to interventions and therapies for the children’s communicative and linguistic development [34,35,36].

Therefore, attention must be paid to the primary caregiver, who carries a significant burden in the family context [22]. Family-focused EI provides support not only to the child but to all family members [37,38]. Family resilience describes the ability of family members to cope with, sustain, or adapt to stressful situations.

Promoting family resilience is recognized in the educational sphere [39,40,41,42,43]. Moreover, enabling families to acquire skills and strategies to cope with and overcome difficulties in educational settings has a positive impact on academic performance [44,45,46] and the infant’s emotional well-being [46,47,48]. By promoting this resilience, family–school bonds are strengthened, creating an effective partnership that fosters academic success and the holistic development of students [49,50]. Children with communication and language disorders require interdisciplinary interventions, with a higher risk of social, behavioral, and educational issues [51,52,53]. Early detection and intervention allow language and communication challenges to be identified and addressed as soon as possible, implementing strategies and supports in scholar settings school environment [23,38,52,54]. This only promotes children’s language and communication, but also active participation in the classroom, academic performance, and emotional well-being [49,50].

Although EI in the school context equips educators with appropriate tools and approaches to support the learning and inclusion of these students, there is no clear evidence regarding parents’ perception in this regard, as well as the needs or demands they identify concerning their children in AT interventions. At the same time, despite the benefits of these treatments, it is not known how parental resilience and stress can influence these interventions, nor how having a family history of these disorders may condition AT interventions [22,26,40].

Resilience development is influenced by both individual characteristics and personal strengths as well as by environmental aspects [55], including family and social support networks (friends, educational community, neighborhood communities, professionals, support groups, or organizations) [27,31], contributing to resilience building for concerned individuals [56]. As a hypothesis, it is suggested that there will be a significant association between the parental resilience and stress levels of these children’s parents and whether they have received previous treatment, as well as between their history of communication and language disorders, resilience, and parental stress. Therefore, this study aims to assess parents’ perceptions of their experiences, needs, and demands of children with language or communication disorders attending ordinary educational centers and who are receiving EI, and the influence of previous treatments on parental resilience and the relationship between family history of communication and language disorders, resilience, and parental stress were examined. 

## 2. Materials and Methods

### 2.1. Study Design and Ethics Considerations

This manuscript reports a descriptive correlational study following the Strobe Statement for cross-sectional studies [57]. This study adhered to the Declaration of Helsinki and its updates [58], and it was approved by the Committee on Bioethics of the University of Burgos (IR12/2018). Subjects’ participation was voluntary with informed consent, and data were encrypted and stored by the research staff.

### 2.2. Participants and Procedure

The following eligibility criteria were established in this study: (1) having a first-born child; (2) aged between 3 and 6 years; (3) with a clinical diagnosis of communication and language disorder; (4) receiving EI treatment; and (5) enrolled in a regular education center.

A non-probability selection technique based on convenience sampling was used to collect the participants [59]. EI centers in the area were visited in April 2019, where the intended study was explained, and consent was requested from the centers to access families with children presenting communication and language-related disorders. The reference professionals for families were informed; these professionals were typically technicians with university training in Occupational Therapy, Speech Therapy, and Hearing and Language Therapy, serving as intermediaries between the target population and the researchers. They were thus responsible for the in-person data collection. Once the families were informed, and after verifying that the eligibility criteria were met, they filled in the informed consent form and the participation application form. Finally, the booklets collected were coded and filed for later analysis.

Out of the initial 250 questionnaires provided, a total of 233 were collected. However, after review and considering the study’s selection criteria, the total number of participants was 220, resulting in a participation rate of 88%. The questionnaires were excluded due to missing data or errors in the inclusion criteria.

### 2.3. Materials

An ad hoc questionnaire was developed to collect sociodemographic information about the children (age, sex), and the communication and language disorder (history, diagnosis, severity, and intervention).

The Parenting Stress Index-Short Form (PSI/SF) validated in a Spanish sample was used to measure family stress [60]. PSI/SF consists of 36 items divided into three subscales (12 items each) that refer to three factors: parental distress (items 1–12), dysfunctional parent–child interaction (13–24), and difficult child (25–36). The Total Stress score is obtained by adding the 36 items, noting that items 14, 22, and 33 are reversed. The score shows the degree of stress parents experience in carrying out their parenting role. Scores above 90 suggest a clinically significant stress level is being experienced by the parent. The internal consistency of the scale factors was 0.90 for childcare stress and 0.87 for personal distress [60]. 

The Wagnild and Young Resilience Scale (RS) [61,62,63,64,65,66] was used to assess resilience. This tool identifies the degree of individual resilience, understood as a positive personality trait that enhances individual adaptation [67]. It is a Likert-type questionnaire ranging from 1 to 7 points from strongly disagree to strongly agree, with the final score obtained by summing the item scores (range 25–175). Scores below 125 indicate low resilience and above 145 high resilience. The RS has high reliability (α = 0.93) and good indicators of construct validity [63].

### 2.4. Statistical Analysis

Statistical analysis was performed using the SPSS v25 statistical package, including the dependent variables determined by the standardized scales and the independent variables representing parental characteristics.

The Kolmogorov–Smirnov test was used to assess the distribution of the data. Based on the results obtained, decisions were made about the type of statistical tests to be used in the study. First, parametric tests (Student’s *t*-test, ANOVA and Pearson’s correlation) were chosen to analyze differences between groups or variables. These tests were used since the selected variables showed a distribution close to normality and fulfilled other parametric assumptions.

Additionally, data that did not fit normality were analyzed with non-parametric tests (Mann–Whitney U-test and Kruskal–Wallis test) to compare qualitative independent variables and quantitative independent variable relationships, respectively. 

This strategy of combining parametric and non-parametric tests allowed for a comprehensive assessment of the data, taking advantage of the nature of the variables measured and avoiding limitations that could arise from relying solely on a statistical approach [68,69]. By adopting this comprehensive approach, a more complete and rigorous perspective of the study results was obtained, allowing researchers to gain a more accurate understanding of the relationships and differences studied.

## 3. Results

The sociodemographic and descriptive characteristics of the 220 participants who agreed to be part of the study in the different EI centers in the province of Cáceres (Spain), are detailed in Table 1. It can be observed that the predominant gender among the children is male, with 74% compared to female (26%). The majority of the children are between 49 and 54 months old (22.73%), followed closely by those between 55 and 60 months (20%).

On the other hand, considering the clinical characteristics of the sample, Table 1 shows that 90.45% of the total sample had no history of communication or language disorders, while 9.55% did have some history. Similarly, 104 participants (47.27%) reported having some speech-related disorders, 56 participants (25.45%) had language-related disorders, and 26 participants (11.81%) had communication-related disorders. In addition, 34 participants (15.47%) had a diagnosis of “others”, which included other diagnoses that involve communication and language disorders or a lack of knowledge about their child’s diagnosis on the part of the participants, meaning they are aware that their child has a problem related to communication and language but do not know which of the previous categories it belongs to.

Furthermore, it is important to know how many children had received previous treatments to try to reduce these communication and language problems. It was found that 92.27% had not received any previous assistance. Those data could be interesting because if positive results had not been obtained in such treatment, the resilience capacity might have been affected.

As seen in Table 1, the majority of the participants did not have a family history of communication disorders (90.45%), and 92.27% reported not having received prior treatment, indicating that most participants had no previous experience in treating their child’s disorder. After applying the *t*-test, Table 2 displays the results of this analysis between prior treatments and the resilience variable. Significant differences in resilience, as measured by the RS scale, were observed between parents who had attended prior treatments and those who had not (*p* = 0.011), with a lower resilience capacity in those with prior experiences compared to those who had not undergone previous treatment.

Table 3 presents the results of the U-tests for the variable “history of communication and language disorders” in relation to the variables of Resilience (Connor scale), Psychological Distress, Dysfunctional Interaction, and Difficult Child. The results show that only the variable “Dysfunctional Interaction” showed statistical significance (*p* = 0.038), indicating statistically significant differences between the groups regarding the family history of language in relation to dysfunctional interaction. It can be observed that the group with a family history has a higher level of dysfunctional interaction (Mean = 31.38) compared to the group that “No” had a family history (Mean = 30.12). In the rest of the analyzed variables, no statistically significant differences were found between the group with and without family history.

## 4. Discussion

This study aimed to evaluate the perception of parents of children aged 3 to 6 years who attend ordinary educational centers and receive EI, obtaining information about their experience, needs, and demands, and considering the impact of previous treatments on parental resilience and the relationship between family history of communication and language disorders, resilience and parental stress. The results provided valuable information to improve the quality and effectiveness of EI services favoring the holistic development of children and the well-being of families.

Most of the participants had no family history of communication and language disorders, nor had they received previous treatment. A significant relationship was found between previous treatment attendance and parental resilience, as well as between family history and dysfunctional parental interaction with children. These findings highlight the importance of considering resilience and previous experience in managing the disorder when designing interventions in EI settings and in schools to improve families’ adjustment and well-being.

Resilience is essential in the adaptation of the person to traumatic situations in which various aspects act to determine how the subject reacts to convert adversity into an opportunity to overcome and develop new possibilities in their lives [70]. For this reason, we asked what resilience capacity the parent of a child with communication and language problems who attends EI treatment would have if they had previously had the experience of attending another center to treat this difficulty. The results show a significant difference between those who have had previous treatment and their resilience (*p* = 0.011), although all of them show low levels of resilience by achieving scores below 125. This finding is in line with the first studies on the subject, which state that experience can enhance resilience [71,72]. In line with the findings of this study, previous research has supported the idea that parents who have previously received EI treatment may develop greater resilience with their children with language and communication disorders [13,73,74]. Another study supports the relationship between pre-treatment experience and parental resilience, finding that parents who had participated in EI programs had greater confidence in their parenting skills and greater ability to manage stress related to their child’s language and communication disorder [75].

On the other hand, a history of communication and language disorders led to the hypothesis that those with a family history of these types of disorders would show a lower stress index given their experience in coping with the disorder. The results indicate that there is a significant relationship between people with no family history of communication and language disorders and the subscale of the Parental Stress Index Dysfunctional Parent–Child Interaction. This indicates that people with no family history of communication and language disorders are less able to interact with their children. This result is probably due to the stress of caring for the child [59]. Some studies support the results found in this study, suggesting that a family history of communication and language disorders may have an impact on parental stress [31]. They also found that parents with a family history of communication and language disorders had higher levels of stress compared to those with no history. This could be related to the additional demands and parenting challenges associated with previous experience in managing the disorder [31,76]. On the other hand, some studies do not support the results obtained in this study and suggest a more complex relationship between family history and parental stress. The presence of a family history of communication and language disorders was not directly related to parental stress [77,78]. However, they found that other factors, such as social support and coping skills, moderated this relationship.

Moreover, other studies have indicated that having higher levels of resilience is associated with a more benign perception of the aspects surrounding children with some form of communication and language disorders [79,80]. It has also been observed that resilience and the perception of better family functioning are related, resulting in cohesion and adaptability. Some authors reveal that the presence of this capacity in parents allows for a better assessment and a less negative response to their lived situations [81,82].

The practical implications of this study include the need to promote the resilience and well-being of infants with language and communication disorders who attend mainstream schools and receive care in early childhood centers. To achieve this, it is essential to ensure adequate treatment attendance in early childhood centers, providing families with tools and strategies to cope with the challenges associated with these disorders. Furthermore, the collaboration between EI centers and regular educational centers should be strengthened, ensuring the continuity of programs and strategies used in both contexts and promoting effective communication between education professionals. The impact of previous experience in dealing with language and communication disorders on parents’ resilience should also be considered. Emotional support and counseling are needed for those who have had no previous experience, as they may face higher levels of stress and difficulties in interacting with their children. Health and education professionals play a key role in providing coping strategies and parental support.

In addition, it is essential to promote greater awareness and awareness of the specific needs of children with language and communication disorders in mainstream schools. This involves training school staff to understand and adequately address the challenges of these children, implementing curricular adaptations, providing additional support and promoting social inclusion strategies. Finally, the active participation of parents in their children’s educational process should be encouraged by providing them with information, resources, and emotional support. This will enable them to collaborate effectively with teachers and strengthen the intervention process, thus promoting the optimal development of children. These practical implications point to the importance of a comprehensive and collaborative approach in the care of infants with language and communication disorders, both in early childhood centers and in mainstream schools. By implementing these recommendations, care services can be optimized, promoting the well-being and inclusion of these children and their families. Limitations are that convenience sampling was used, which limits the generalizability of the findings. In addition, the procedure for recruiting participants was based on the collaboration of professionals from EI centers, which could introduce biases in the selection of participants. Furthermore, it is important to bear in mind that this study focused on a specific sample of parents of children attending ordinary educational centers and receiving EI in associations in the province of Caceres, Spain. Therefore, the results may be influenced by contextual and cultural factors specific to this population, which limits their generalizability to other geographical regions and more diverse populations. Another limitation relates to the method of data collection, which relied mainly on questionnaires and self-reports. This may introduce biases due to the subjectivity of responses and the possibility of omitting relevant information. It would be advisable to complement the data obtained with other methods, such as direct observations or clinical assessments, to obtain a more complete and objective perspective on the variables studied. This study was based on cross-sectional data, which prevent us from establishing causal relationships between the variables analyzed. It would be of interest to conduct longitudinal research or experimental studies that would allow for a more precise examination of causal relationships and the evolution of variables over time.

## 5. Conclusions

There is a significant association between previous treatment and parents’ resilience, suggesting that previous experience may strengthen their coping capacity. Additionally, most participants did not have a family history of communication and language disorders, which may influence their ability to interact with their children.

## Figures and Tables

**Table 1:** Clinical and demographic characteristics of the study group.

Variables		Total (*n* = 220)
Gender of children	Male	74% (*n* = 163)
	Female	26% (*n* = 57)
Age of children (months)	36–42	5.45% (*n* = 12)
	43–48	12.27% (*n* = 27)
	49–54	22.73% (*n* = 50)
	55–60	20% (*n* = 44)
	61–66	18.18% (*n* = 40)
	67–72	21.36% (*n* = 47)
History of communication and language disorders	With a history 21	9.55% (*n* = 21)
	Without a history 199	90.45% (*n* = 199)
Communication and language disorders	Speech	47.27% (*n* = 104)
	Language	25.45% (*n* = 56)
	Communication	11.81% (*n* = 26)
	Others	15.47% (*n* = 34)
Attendance to other treatments previously	Yes	7.73% (*n* = 17)
	No	92.27% (*n* = 203)

**Table 2:** *t*-test for the analysis of the pretreatment variable.

	Pretreatments	N	Mean	Standard Deviation	*p*
Resilience (Total RS)	Yes	17	111.82	11.091	0.011 *
	No	203	103.35	13.148	

RS: The Wagnild and Young Resilience Scale; * *p* = statistically significant value.

**Table 3:** U-test for the history of family communication and language disorders variable.

Variables	History	N	Mean	Standard Deviation	*p*
Resilience	Yes	21	103.61	13.078	0.875
	No	199	104.05	13.217	
Psychological distress	Yes	21	107.14	9.758	0.803
	No	199	105.15	7.707	
Dysfunctional interaction	Yes	21	31.38	6.119	0.038 *
	No	199	30.12	5.942	
Difficult child	Yes	21	58.71	7.198	0.662
	No	199	54.64	10.288	

* *p* = statistically significant value.

## Data Availability

The data presented in this study are available on request from the corresponding author. The data are not publicly available due to the policy of the Doctorate in Education Program of the University of Burgos.
